# Niclosamide rescues microcephaly in a humanized *in vivo* model of Zika infection using human induced neural stem cells

**DOI:** 10.1242/bio.031807

**Published:** 2018-01-15

**Authors:** Dana M. Cairns, Devi Sai Sri Kavya Boorgu, Michael Levin, David L. Kaplan

**Affiliations:** 1 Department of Biomedical Engineering, Tufts University, Medford, MA 02155, USA; 2 Department of Biology, Tufts University, Medford, MA 02155, USA; 3 Allen Discovery Center, Tufts University, Medford, MA 02155, USA

**Keywords:** Zika virus, Chick embryo, Neural stem cells, Microcephaly

## Abstract

Zika virus (ZIKV) is a mosquito-transmitted flavivirus with a causative link to microcephaly, a condition resulting in reduced cranial size and brain abnormalities. Despite recent progress, there is a current lack of *in vivo* models that permit the study of systemic virus on human neurons in a developing organism that replicates the pathophysiology of human disease. Furthermore, no treatment to date has been reported to reduce ZIKV-induced microcephaly. We tested the effects of ZIKV on human induced neural stem cells (hiNSCs) *in vitro* and found that infected hiNSCs secrete inflammatory cytokines, display altered differentiation, and become apoptotic. We also utilized this *in vitro* system to assess the therapeutic effects of niclosamide, an FDA-approved anthelminthic, and found that it decreases ZIKV production, partially restores differentiation, and prevents apoptosis in hiNSCs. We intracranially injected hiNSCs into developing chicks, subjected them to systemic ZIKV infection via the chorioallantoic membrane (CAM), a tissue similar in structure and function to the mammalian placenta, and found that humanized ZIKV-infected embryos developed severe microcephaly including smaller crania, decreased forebrain volume and enlarged ventricles. Lastly, we utilized this humanized model to show that CAM-delivery of niclosamide can partially rescue ZIKV-induced microcephaly and attenuate infection of hiNSCs *in vivo*.

This article has an associated First Person interview with the first author of the paper.

## Abstract (precis)

**Summary:** Injecting hiNSCs into developing chick brains and systemically infecting with ZIKV produces embryos with severe microcephaly. Using this humanized model we show that niclosamide rescues ZIKV-induced microcephaly and decreases infection of hiNSCs *in vivo*.

## INTRODUCTION

Zika virus (ZIKV) is a mosquito-transmitted flavivirus that has been the cause of recent public health concern mostly due to its causative link to microcephaly, a congenital birth defect in which babies are born with abnormally small heads and deficits in brain development. With recent epidemics in Central and South America (Soares de Oliveira-Szejnfeld et al., 2016), there is an urgent need to develop physiologically relevant ZIKV-infection models that can be used to study the pathophysiology of the disease and to identify new potential therapeutic agents. Several groups have generated *in vitro* models using relevant human cell types including induced pluripotent stem cell (iPSC)-derived neural progenitors (Tang et al., 2016), neurospheres and brain organoids (Dang et al., 2016; Gabriel et al., 2017; Garcez et al., 2016), primary fetal neural cells (Liang et al., 2016; Onorati et al., 2016) and endothelial cells (Liu et al., 2016). These *in vitro* studies have helped to elucidate some of the molecular mechanisms contributing to the pathogenesis of ZIKV infection. For example, it was recently shown that ZIKV-infected cranial neural crest cells (CNCCs) secrete multiple factors that may have a paracrine effect on surrounding tissues during development (Bayless et al., 2016). This is important because most cases of microcephaly involve not only brain deformities but also craniofacial abnormalities, suggesting that the detrimental effect of ZIKV is not restricted to cells of neural lineage. It was found that ZIKV-infected CNCCs do not become apoptotic but secrete leukemia inhibitory factor (LIF) and vascular endothelial growth factor (VEGF), paracrine factors which cause neurospheres to become migratory and display neurite projections. This suggests that these ZIKV-induced factors can have a profound effect on neurogenesis (Bayless et al., 2016), which may ultimately contribute to the microcephalic phenotype. While these *in vitro* studies have helped to understand some of the various pathways involved in ZIKV pathogenesis, they do not recapitulate the microcephalic phenotype seen in patients.

Multiple *in vivo* mouse models have been developed, however, these models often require the use of immunocompromised mouse strains (Cugola et al., 2016) or alternatively have utilized various methods of non-systemic infection including the direct inoculation of ZIKV into the uterine wall of pregnant, immunocompetent mice (Vermillion et al., 2017) and ZIKV injection of embryonic brains for *ex vivo* culture (Li et al., 2017, 2016); have a shortened window of prenatal exposure time (Miner et al., 2016); and perhaps most significantly, most mouse models do not truly recapitulate classical human microcephaly in reduced cranial size (Lazear et al., 2016). Importantly, to date, no one has combined the two platforms to incorporate human neural cells into an *in vivo* organism to understand the effects of systemic virus and drug treatment on human neurons.

Here, we utilized human induced neural stem cells (hiNSCs), which have been previously characterized and described (Cairns et al., 2016), as a basis for both our *in vitro* and *in vivo* models of ZIKV infection. Stable hiNSC lines are established through the direct reprogramming of neonatal human fibroblasts. hiNSCs rapidly differentiate into beta III tubulin (Tuj1)-positive neurons independently of media composition, making them ideal for complex co-cultures and various drug screening applications. Furthermore, these cells are particularly useful for this study in that hiNSCs injected into the developing neural tubes of a chick embryo migrate, differentiate and integrate into both the central and peripheral nervous systems; maintaining their neuronal phenotype even in non-neuronal microenvironments (Cairns et al., 2016)

We first analyzed the *in vitro* effects of ZIKV on hiNSCs. We demonstrate that infected hiNSCs secrete ZIKV, inflammatory cytokines and growth factors; display altered differentiation; and become highly apoptotic. We also utilize this *in vitro* system to assess the therapeutic effects of Niclosamide (NIC), an FDA-approved anthelminthic recently identified in an anti-ZIKV drug screen, and found that it reduces ZIKV production, partially restores differentiation, and prevents apoptosis in hiNSCs.

After establishing the *in vitro* effects of ZIKV, we wanted to develop a physiologically relevant *in vivo* model incorporating hiNSCs. We selected the chick embryo for a variety of reasons. First, the ability to inject human neural stem cells directly into the developing brain is not amenable to embryonic rodent systems. Further, because chicks are independent upon hatching, their brain development occurs relatively rapidly (Lundgren et al., 1995). The chick telencephalon develops 1-2 weeks faster than in rodent models (Schneider and Norton, 1979). This increased rate of avian maturation is desirable as it more accurately reflects human physiology in that both newborns and hatchlings have well-developed brains at partus (Zhou et al., 2015). In addition, some mouse models to date have had to specifically utilize immunocompromised strains to allow for ZIKV infection, as common strains systemically infected with ZIKV have no microcephalic phenotype (Cugola et al., 2016). The innate immune system of the chick embryo does not function until around E14 (Kain et al., 2014) suggesting that this *in vivo* system provides a naturally immunodeficient model system suitable for both human xenografts as well as viral infection (Bjornstad et al., 2015). We intracranially injected hiNSCs into developing chick embryos, subjected them to systemic ZIKV infection and found that humanized ZIKV-infected embryos developed severe microcephaly consisting of significantly reduced cranial size, decreased forebrain volume and enlarged ventricles. Lastly, we utilized this humanized model for subsequent drug testing and found that NIC can partially rescue ZIKV-induced microcephaly and attenuate infection of hiNSCs *in vivo*.

Our findings are of particular importance given both the immediate public health concern as well as the novelty of the study. Our humanized *in vivo* model system is unique in that it allows for the study of systemic virus on human neurons in a developing organism. We show that in the absence of intracranially injected hiNSCs, the resulting embryonic phenotype is unremarkable. In addition, our method of ZIKV delivery onto the chorioallantoic membrane (CAM) is physiologically relevant in that it is systemic and spread via tissues similar in structure and function to the developing placenta. Furthermore, no therapeutic to date has been reported to reduce ZIKV-induced microcephaly in animal studies. We utilize a dosage comparable to those used clinically for parasite treatment, and deliver the drug in a method analogous to human transplacental delivery. We also show that treating with NIC at high doses, even in the absence of ZIKV infection, has no discernible effects on embryonic development, suggesting that NIC may not be teratogenic in other organisms, including humans. In the future, this humanized *in vivo* model of Zika-induced microcephaly using hiNSCs could be utilized to validate other treatments and could be further adapted to understand the pathophysiology of other infectious agents such as cytomegalovirus (CMV) or *Toxoplasma gondii*, which are known to disrupt brain development upon exposure during pregnancy (Cordeiro et al., 2015).

## RESULTS

### hiNSCs are highly infectable by ZIKV which can be attenuated by NIC *in vitro*

**Fig. 1.**
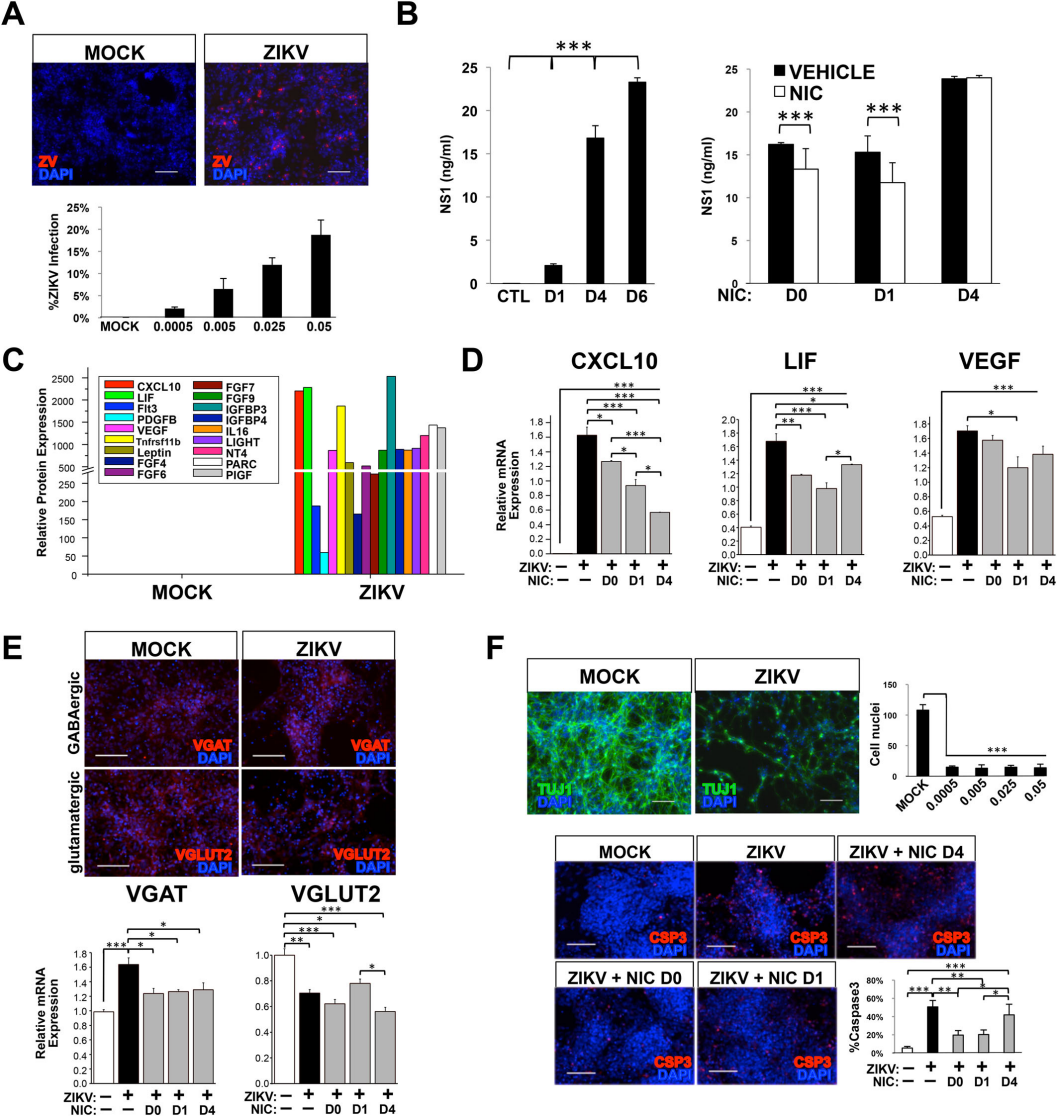
**hiNSCs secrete virus, display an altered secretome and differentiation profile, and undergo apoptosis in response to ZIKV infection, which can be attenuated by Niclosamide treatment *in vitro*.** (A) Infection rate of hiNSCs at different MOIs as determined by immunostaining against flavivirus envelope protein. Scale bar: 100 µM. (B) ELISA assay of secreted ZIKV NS1 protein in cell culture supernatants indicates that hiNSCs secrete increased ZIKV NS1 over time, which can be attenuated by pre- or concurrent NIC treatment. (C) Cytokine array data showing that hiNSCs secrete a variety of cytokines and growth factors. (D) qRT-PCR data indicating that NIC inhibits the expression of CXCL10, LIF and VEGF to varying degrees. (E) ZIKV affects the differentiation profile of hiNSCs. ZIKV infection causes hiNSCs to express higher levels of VGAT and lower levels of VGLUT2, and NIC partially restores the expression of VGAT to control levels. Scale bar: 100 µM. (F) ZIKV induces massive cell death in hiNSCs. By day 10, most hiNSCs are dead regardless of starting MOI. Panel shows immunostaining of mock or ZIKV-infected hiNSCs (MOI 0.0005). Pre- or concurrent NIC treatment can partially restore rate of Caspase3 expression to normal. Scale bar: 100 µM. *P≤0.05, **P≤0.01, ***P≤0.001; as determined by one-way ANOVA with post hoc Tukey test. Error bars show mean±s.d.

To first test the effects of ZIKV on hiNSCs *in vitro*, hiNSCs were exposed to the ZIKV^AF^ strain MR-766 (MOI 0.0005–0.05), which shares 87-90% sequence similarity with Brazilian isolates from microcephalic patients (Calvet et al., 2016) to determine their infectability. After 4 days, ZIKV was detected by immunostaining against the flavivirus envelope protein (Fig. 1A). We also analyzed the expression of secreted ZIKV protein non-structural protein 1 (NS1) to detect the presence of ZIKV secreted by cells *in vitro*. While NS1 protein is secreted independently of ZIKV, it is often used as a surrogate marker for viral replication and production (Xu et al., 2016). We also confirmed infection by quantifying ZIKV RNA copy in hiNSCs by qRT-PCR analysis (Fig. S1). Supernatants from infected hiNSCs were collected at D1, D4 and D6 and analyzed by ELISA for the NS1 protein. This timecourse demonstrated that the NS1 protein present in the supernatant increases over time, suggesting that hiNSCs are actively secreting ZIKV (Fig. 1B).

We next wanted to determine the effect of niclosamide (NIC), an FDA-approved anthelmintic drug that was recently discovered to have anti-ZIKV effects *in vitro* in glioblastoma SNB-19 cells (Xu et al., 2016), on infection in hiNSCs. We specifically selected this drug as it is a category B drug, which has shown no teratogenic or toxic effects to fetuses in animal studies. We first determined a suitable NIC concentration based on initial *in vitro* studies (Fig. S2). While the precise molecular mechanism by which NIC decreases ZIKV production remains somewhat elusive, it has been determined that timing of NIC treatment *in vitro* can be critical to the efficacy of the drug in preventing ZIKV infection (Xu et al., 2016). To test this, we treated hiNSCs with 0.5 µM NIC at different timepoints – either one day before (D0), concurrently (D1) or 3 days after infection (D4). hiNSCs receiving pre- or concurrent NIC treatment upon infection, significantly decreased ZIKV production as determined by NS1 ELISA assays performed on hiNSC supernatants (Fig. 1B). Conversely, hiNSCs treated with NIC 3 days post-infection were unable to decrease NS1 levels in response to treatment. Therefore, we demonstrated that pre- or concurrent NIC treatment is effective in attenuating ZIKV infection in hiNSCs *in vitro*.

### ZIKV-infected hiNSCs exhibit a unique secretome and differentiation profile that can be partially restored with NIC treatment

We also sought to understand some of the molecular changes that occur in ZIKV-infected hiNSCs. We performed a cytokine array on supernatants from mock and ZIKV-infected hiNSCs grown for 4 days. Interestingly, we found that ZIKV-infected hiNSCs secrete a variety of cytokines and growth factors known to be involved in multiple cell processes (Fig. 1C). For example, C-X-C motif chemokine 10 (CXCL10), a pro-inflammatory cytokine known to be upregulated in brains of mice infected with rabies virus (Chai et al., 2015), as well LIF and VEGF, two factors previously found to be secreted by ZIKV-infected neural crest cells (Bayless et al., 2016), were found to be dramatically upregulated in ZIKV-infected hiNSCs at both the protein and mRNA level (Fig. 1C-D). Furthermore, we found that that NIC treatment was able to significantly decrease their relative expression to varying degrees (Fig. 1D).

In addition to understanding the potential paracrine effects of ZIKV on hiNSCs, we also wanted to understand the effects of ZIKV on hiNSC differentiation. hiNSCs have been shown to spontaneously differentiate into both inhibitory GABAergic (vesicular GABA transporter, VGAT+) and excitatory glutamatergic (Vesicular Glutamate Transporter, VGLUT2+) neurons in under 2 weeks (Cairns et al., 2016). Interestingly, we found that ZIKV-infected hiNSCs (MOI=0.0005) expressed significantly higher levels of VGAT and lower levels of VGLUT2 than mock-infected cells, suggesting that ZIKV-hiNSCs are more predisposed to becoming GABAergic neurons (Fig. 1E). Intriguingly, NIC treatment significantly reduces VGAT upregulation in ZIKV-infected hiNSCs, however it cannot rescue VGLUT2 expression to that of mock-infected hiNSCs.

### Pre- or concurrent NIC treatment prevents ZIKV-induced hiNSC apoptosis *in vitro*

Lastly, we wanted to determine if ZIKV affected cell viability in hiNSCs. We cultured mock or ZIKV-infected hiNSCs, and immunostained for pan-neuronal marker beta-III tubulin (Tuj1) to visualize neurite extensions. After 10 days in culture, most cells had died, even at the lowest MOI tested (0.0005). We also wanted to ascertain if NIC could prevent apoptosis. Mock or ZIKV-infected were dosed with vehicle or NIC at various timepoints, fixed at D6, and immunostained for apoptotic marker cleaved Caspase 3 (Csp3). Interestingly, we found that either pre- or concurrent NIC treatment significantly reduced the percentage of Csp3+ cells, but post-treatment did not (Fig. 1F).

Taken together, our *in vitro* data suggest that hiNSCs are highly infectable and responsive to ZIKV infection. Furthermore, pre- and/or concurrent treatment with NIC is able to attenuate virus production, reduce certain ZIKV-induced inflammatory mediators, partially rescue altered neuronal differentiation, as well as reduce cell apoptosis in hiNSCs. These *in vitro* studies allowed us to determine the timing of the drug treatment regimen for subsequent *in vivo* studies.

### *In vivo* injection of ZIKV-infected hiNSCs into developing chick brains causes severe microcephaly

**Fig. 2.**
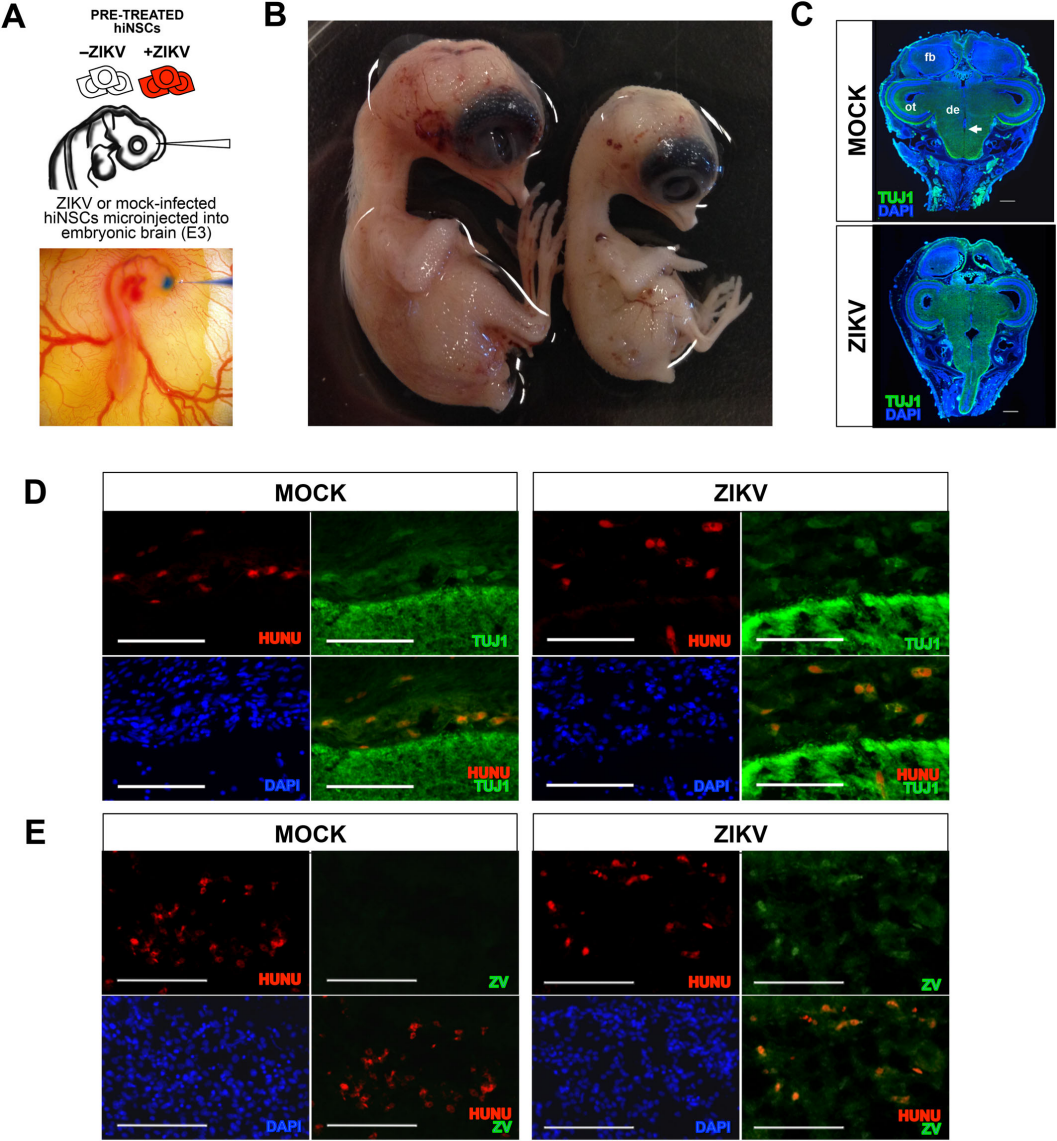
**Intracranial injection of ZIKV-infected hiNSCs into chick embryos results in microcephaly.** Injection of ZIKV-infected hiNSCs results in microcephaly. (A) Schematic and image of intracranial injection. (B) Embryos injected with mock- (left) or ZIKV-infected (right) hiNSCs 10 days post-injection. (C) Coronal sections showing pan-neuronal Tuj1 immunostaining showing morphological characteristics. fb, forebrain; ot, optic tectum (midbrain); de, diencephalon; arrow, ventricle. Scale bar: 1 mM. (D) Immunostaining indicating presence of hiNSCs (HuNu+) in the developing brain of E12 chick embryos, which are also positive for Tuj1. Scale bar: 100 µM. (E) ZIKV-infected hiNSCs are also positive for anti-flavivirus marker (ZV). Scale bar: 100 µM.

Our lab has previously shown that injection of hiNSCs into the developing neural tubes of a chick embryo allows for the incorporation into both the central and peripheral nervous system (Cairns et al., 2016). To establish our humanized model of ZIKV infection, we used a similar approach and microinjected mock or ZIKV-infected hiNSCs into the developing encephalon, then allowed the embryos to develop *in ovo* for approximately 10 days (Fig. 2A). The model is such that at the early stage of development during which intracranial microinjection occurs, the number of injected hiNSCs relative to the number of resident chick cells is relatively high, and they are still proliferative. As they migrate and differentiate they stop proliferating, so by E12-13 their population relative to resident chick cells is much lower. Strikingly, embryos receiving intracranial injections of ZIKV-infected hiNSCs exhibited a dramatic reduction in head size as well as an ocular phenotype (Fig. 2B) reminiscent of those previously described in microcephalic patients (de Paula Freitas et al., 2016), as well as a previously described *in vivo* mouse model (Cugola et al., 2016). Because microcephaly affects tissues outside the brain, we sectioned through entire intact embryo heads to preserve the native structure of the surrounding tissue. Coronal cryosections immunostained for pan-neuronal Tuj1 show the resulting histology of the differentially treated embryos (Fig. 2C; for visualizing plane of section through developing brain Fig. S3). Furthermore, we demonstrated that the resulting hiNSCs remained neuronal in phenotype by co-staining for human nuclear antigen (HuNu) (Fig. 2D) and that ZIKV was detectable in those hiNSCs present in the brain (Fig. 2E).

### Intracranial injection of hiNSCs followed by systemic ZIKV infection causes microcephaly which can be prevented by NIC treatment *in vivo*

**Fig. 3.**
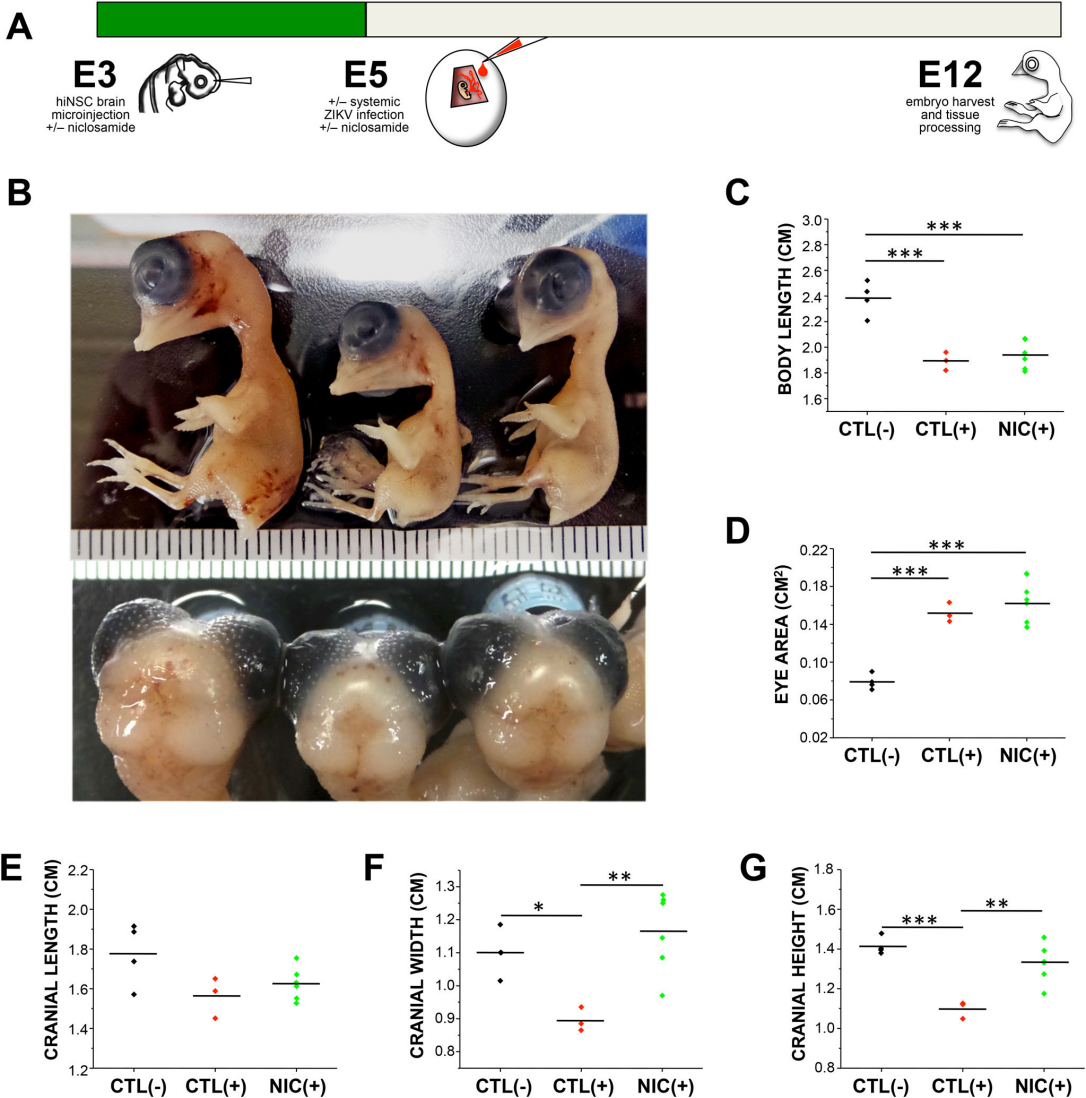
**Systemic Niclosamide treatment can partially rescue ZIKV-induced reduction in cranial size in a humanized *in vivo* model.** (A) Schematic diagram showing experimental timeline of injection of hiNSCs as well as systemic ZIKV infection and NIC treatment. Briefly, embryos were injected with hiNSCs intracranially and treated with the first dose of either vehicle or NIC via the CAM at E3. At E5, embryos were subjected to systemic ZIKV infection as well as the second dose of either vehicle or NIC via the CAM, and all embryos were harvested at E12. (B) Image of E12 embryos after treatment (left to right: Mock plus vehicle, ZIKV plus vehicle, ZIKV plus NIC). Quantification of body length (C), eye area (D) as well as morphological features of the cranium including length (E), width (F) and height (G) across treatments. *P≤0.05, **P≤0.01, ***P≤0.001; as determined by one-way ANOVA with post hoc Tukey test.

While our initial approach of injecting ZIKV-infected hiNSCs into embryonic brains resulted in a clinically relevant and striking phenotype, we wanted to further adapt the model to more accurately reflect the mode of transmission in humans and to incorporate an appropriate window for therapeutic treatment. For these reasons, we slightly modified our approach as follows (Fig. 3A). Briefly, we still injected hiNSCs intracranially at E3 except that all hiNSCs were healthy and uninfected. Based on our *in vitro* data, we also pre-treated embryos systemically at this timepoint by applying either vehicle or NIC directly onto the developing CAM. As we wanted our humanized system to best reflect the effects of potential clinical treatment options, we selected a specific dosage that was within reasonable range of previously used dosages (70-150 mg/kg) in clinical studies of NIC for anti-parasitic treatment (Hayes and Laws, 1991). An appropriate dosage for *in vivo* treatment was determined as follows. The weight of an E3 chick embryo is approximately 20 mg (Levy and Palmer, 1940). Dosage per egg was 1 µg per egg, which corresponds to a dosage of 50 mg/kg. At E5, we used the CAM method to systemically infect with ZIKV as well as to apply a second dose of NIC (based on effects of both pre- and concurrent NIC treatment *in vitro*). We allowed the embryos to develop until E12 at which point they were sacrificed and analyzed.

We found that embryos infected with ZIKV showed a decreased survival rate compared to mock-infected embryos, and that NIC restored this survival rate almost back to normal (Table S1). We quantified the phenotypes of resulting embryos (Fig. 3B).

We also quantified phenotypic changes in body length and found that both ZIKV- and ZIKV+NIC-treated embryos had significantly shorter body lengths compared to mock-infected embryos (Fig. 3C). Interestingly, we also observed an ocular phenotype in ZIKV-infected embryos (Fig. 3D). We quantified eye area and found that ZIKV-treated embryos exhibited larger ocular openings. We also found that there were significant differences in cranial length, width, and height (Fig. 3E-G; Fig. S3 for dimensions of analysis), and that NIC was able to significantly rescue both cranial width (Fig. 3F) and height (Fig. 3G). These *in vivo* experiments have been repeated independently three times with a similar trend resulting from each experiment. The data in this figure is the specific result from one of those experiments.

### NIC treatment attenuates ZIKV infection and partially restores altered differentiation of hiNSCs *in vivo*

**Fig. 4.**
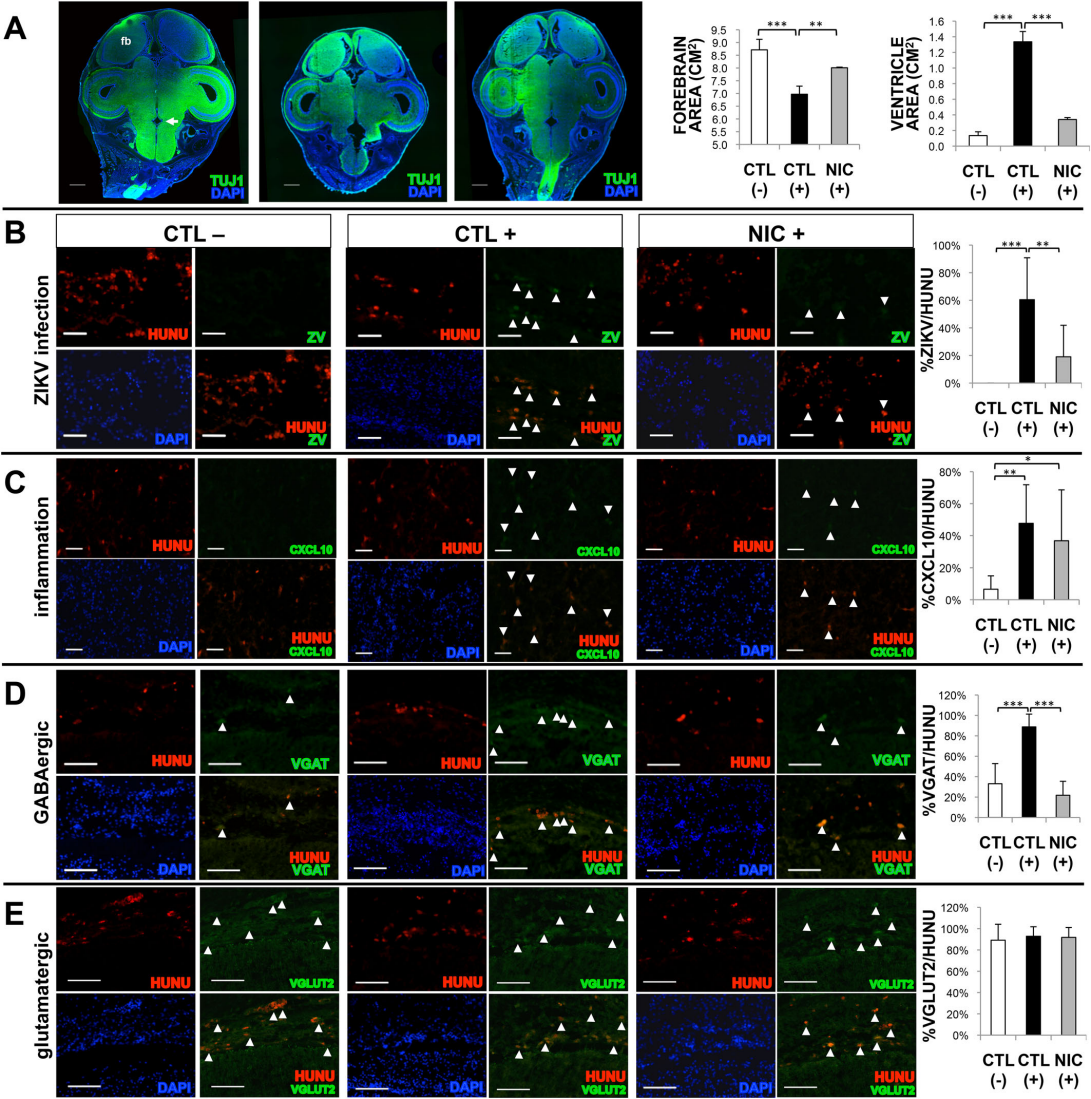
**Niclosamide treatment reduces ZIKV infection and altered differentiation of hiNSCs *in vivo*.** (A) Tuj1 immunostaining through the intact cranium. fb, forebrain; arrow, 4th ventricle. Scale bar: 1 mM. Quantification of forebrain and 4th ventricle area across treatments. (B) Immunostaining showing co-localization of HuNu and ZV to quantify the number of ZIKV-infected hiNSCs. Scale bar: 50 µM. (C) Immunostaining showing co-localization of HuNu and CXCL10 with quantification. Scale bar: 50 µM. (D,E) Immunostaining showing co-localization of HuNu with either VGAT (D) to indicate the presence of GABAergic neurons or VGLUT2 (E) which indicates glutamatergic neurons, with respective quantification. Scale bar: 100 µM. *P≤0.05, **P≤0.01, ***P≤0.001; as determined by one-way ANOVA with post hoc Tukey test. Error bars show mean±s.d.

Lastly, we wanted to understand the fate of the injected hiNSCs in response to systemic ZIKV infection and/or NIC treatment. As in previous experiments, we generated coronal sections of these embryos and subjected them to Tuj1 immunostaining to visualize the morphological differences across treatments. Significant differences in forebrain area as well as area of the 4th ventricle (the central cavity of the hindbrain) were found in ZIKV-infected embryos, and NIC was able to significantly rescue them (Fig. 4A). Interestingly, similar findings such as abnormal cerebral volume, brainstem malformations, and enlargement of the fourth ventricle have all been reported previously in clinical ZIKV-induced microcephalic patients (Soares de Oliveira-Szejnfeld et al., 2016), suggesting that perhaps NIC treatment may also be able to prevent some of these abnormalities in humans.

We also stained for flavivirus envelope protein and HuNu to co-localize infection with hiNSCs. Importantly, we found that NIC was able to significantly reduce the percentage of ZIKV+ human cells (Fig. 4B). We also assayed for expression of CXCL10, the previously identified pro-inflammatory marker found to be upregulated in ZIKV-infected hiNSCs. We found that CXCL10 was significantly upregulated in ZIKV-hiNSCs, but that this expression could not be rescued by NIC treatment (Fig. 4C).

Our previous work has shown that hiNSCs have the capacity to differentiate into both inhibitory and excitatory neurons upon injection *in ovo* (Cairns et al., 2016). Based on this as well as our *in vitro* data, we wanted to determine if ZIKV-infected hiNSCs are also predisposed to becoming specific neuronal subtypes *in vivo*. We selected regions from the midbrain where there was relatively high endogenous expression of markers of specific subtypes of neurons in the surrounding resident chick tissue, and used a similar location for all embryos analyzed. We analyzed the percentage of HuNu+ cells that were also positive for either VGAT (GABAergic) (Fig. 4D) or VGLUT2 (glutamatergic) neurons (Fig. 4E). Interestingly, while we did not see a significant difference in the percentage of VGLUT2+hiNSCs in brain tissue across all treatments, we found that a significantly higher percentage of VGAT+hiNSCs were found in brains of ZIKV-infected embryos compared to mock-infected and NIC+ZIKV-infected embryos.

## DISCUSSION

This study utilized hiNSCs for the generation and subsequent drug testing of a humanized *in vivo* model of ZIKV-induced microcephaly. We first analyzed the *in vitro* effects of ZIKV on hiNSCs. We found that infected hiNSCs secrete ZIKV, inflammatory cytokines and growth factors; display altered differentiation; and become highly apoptotic. We also tested the potential therapeutic effects of NIC, an FDA-approved anthelminthic recently identified in an anti-ZIKV drug screen. We found that NIC treatment significantly decreased ZIKV production and partially restored neuronal differentiation of hiNSCs, however, it was unable to rescue levels of certain secreted factors back to those of mock-infected samples. NIC treatment resulted in significant decreases in both ZIKV-induced CXCL10 and LIF expression, however, it did not have a substantial effect on reducing the expression of VEGF. VEGF has been shown to induce increased blood brain barrier (BBB) permeability, and conversely has a prospective neuroprotective role in its ability to promote angiogenesis and oxygenation to damaged nervous tissues (Mackenzie and Ruhrberg, 2012). Given that VEGF has been shown to have both positive and negative roles on neurogenesis and response to injury, the minimal effect of NIC on its expression may be inconsequential.

While the goal of our study was to incorporate a human neural component into a developing organism *in vivo* for the purpose of studying systemic ZIKV infection and potential treatment strategies, we did not anticipate that there would be minimal effects of ZIKV on this avian system in the absence of hiNSCs. It was exciting that our humanized *in vivo* ZIKV-infection model induced microcephaly, however, it was also important to determine if endogenous chicken cells were also infectable and responsive to ZIKV. It was previously reported that chicken cells cannot be infected by flaviviruses (Way et al., 1976), however, embryonated chicken eggs have since been used for isolation of other flaviviruses such as West Nile (Crespo et al., 2009). We first assayed infectability of embryonic chick cortical neurons and dermal fibroblasts relative to hiNSCs by exposing them to the same MOI for 4 days in culture (Fig. S4). We demonstrated that chick cells do appear to be somewhat infectable, but at a dramatically lower rate than that of hiNSCs. Furthermore, unlike hiNSCs, these chick-derived cell types do not appear to be susceptible to ZIKV-induced cell death.

Importantly, our study is not the first to utilize the chick embryo for Zika-related studies. A recent study found that injection of ZIKV into the amnion of E2.5 or E5 chick eggs resulted in an increase in mortality (Goodfellow et al., 2016), however, there was no significant change in cranial size or brain volume reported in surviving ZIKV-treated embryos. The only significant morphological difference between sham and ZIKV-infected embryos was a larger volume of lateral ventricles as determined by nonplanar *in ovo* images captured by magnetic resonance imaging (MRI). Furthermore, the study did not test any therapeutics in this model. We performed similar experiments to systemically infect embryos in the absence of injected hiNSCs. Interestingly, when we infected uninjected chick embryos with ZIKV the resulting phenotype was unremarkable in comparison to that of ZIKV-infected embryos pre-injected with hiNSCs (Fig. S5). This could be due at least in part to the distinct effect of ZIKV on the secretome of infected hiNSCs.

While our humanized *in vivo* model resulted in severe microcephaly consisting of decreased cranial size and various brain abnormalities, it is also important to address the ZIKV-induced reduction in body length. While microcephaly is the most obvious birth defect associated with prenatal Zika infection in humans, it is important to note that Zika infection in pregnant women has also been shown to lead to other disorders including ocular anomalies (Ventura et al., 2016a,b), as well as fetal growth restriction and demise (Oliveira Melo et al., 2016; Rasmussen et al., 2016; Sarno et al., 2016). Furthermore, there have only been a few *in vivo* studies to date that have described any visibly discernible Zika-induced microcephaly as demonstrated by a clear reduction in cranial size (Cugola et al., 2016; Shao et al., 2016). Currently, there are few (if any) *in vivo* animal models of Zika-induced microcephaly in which decreased cranial size is not accompanied by some obvious reduction in body size.

In this study specifically, our model is generated by way of injecting hiNSCs directly into the developing encephalon, which at this early developmental stage, still makes up the anterior neural tube extending throughout the length of the embryo. While our injections are mostly localized to the cranial portion of the neural tube, we hypothesized that some of these hiNSCs potentially migrate throughout the neural tube and contribute to the peripheral nervous system as shown previously (Cairns et al., 2016). We injected either mock- or ZIKV-infected hiNSCs into E3 embryos and allowed them to develop until E7. We cryosectioned the still-intact neural tube and performed immunostaining to detect the presence of hiNSCs that had migrated throughout the neural tube toward peripheral tissues (Fig. S6). If highly responsive hiNSCs are also present in the developing spinal cord and/or peripheral nervous system, it is possible that they are contributing to the phenotypic differences in body length. It is also possible that the various growth factors and cytokines secreted by ZIKV-infected hiNSCs could potentially enter the bloodstream eliciting independent systemic effects. The observation that ZIKV-infected embryos without injected hiNSCs do not exhibit any discernible changes in body length provides potential support of this hypothesis.

Upon further analysis of tissues harvested from treated humanized embryos, we found that NIC treatment was able to partially restore ZIKV-induced changes in forebrain area, ventricle enlargement and hiNSC differentiation *in vivo*. Moreover, we found that NIC treatment was able to restore the relative percentage of VGAT+ hiNSCs to control levels. This is interesting from a developmental standpoint in that aberrations in GABAergic neuron development have been implicated in a variety of neurodevelopmental disorders such as epilepsy (Bozzi et al., 2012) and autism (Coghlan et al., 2012). While these observations are striking, future studies in patients will be helpful to better understand this altered differentiation pattern in response to ZIKV. While aberrant GABAergic signaling has been linked to various neurodevelopmental disorders (Ramamoorthi and Lin, 2011), it remains to be seen if it is also impaired in clinical cases of ZIKV-induced microcephaly.

Importantly, NIC was not able to fully alleviate all ZIKV-induced effects including ocular malformations and increased levels of inflammation in engrafted hiNSCs. NIC treatment was unable to significantly rescue ZIKV-induced increase in eye area, but this could also be due to timing of NIC treatment. Eyelid development does not begin until E7 in the chick, which is 2 days after systemic ZIKV infection and the second dose of NIC. While it is possible that NIC treatment reduces the initial viral load, after several days without subsequent NIC treatment, residual ZIKV present in hiNSCs in the developing eye may begin to produce more virus and potentially more localized cytokines (Ojeda et al., 2013). We also found that the inflammatory cytokine, CXCL10, was significantly upregulated in ZIKV-hiNSCs, but that this expression could not be rescued by NIC treatment. This observation may also be due to timing of NIC treatment. Our *in vitro* data suggested that sustained NIC treatment might be more effective in reducing CXCL10 expression, however, our *in vivo* regimen of drug treatment was only at E3 and E5.

The NIC dosage selected for this study was in the lower range of doses used clinically for treatment of parasites, and may need to be increased to completely eradicate ZIKV in this model system. It also seems plausible that continued NIC treatment past E5 could more efficiently rescue the microcephalic phenotype induced by ZIKV treatment. Future studies could focus on the effects of increased or prolonged NIC dosage. Since our overall goal was to establish an *in vivo* system that could be used to study systemic viral infection on human neurons in a developing organism and to screen for potential therapeutics, we did not feel that it was necessary to optimize the *in vivo* activity of this particular drug.

While these results are striking, in order for a drug to be considered safe whether for treatment or prophylactically in pregnant women, it is also important to assess whether NIC has any teratogenic effects in the absence of ZIKV. Uninfected chick embryos were treated with vehicle, the previous NIC dosage or 5× the previous NIC dosage following the same treatment regimen (Fig. S7). NIC treatment alone has no discernible effects on embryonic development, suggesting that NIC may not be teratogenic in other organisms, including humans.

In summary, our results show that NIC can partially rescue ZIKV-induced microcephaly and can attenuate infection of hiNSCs *in vivo*. While vaccines for ZIKV are currently in development (Muthumani et al., 2016; Richner et al., 2017), an extensive amount of time is required to clinically validate their efficacy and safety. There is an urgent need to treat potentially affected fetuses and prophylactically treat pregnant mothers in regions in which the potential exposure risk is very high. Given that NIC is an FDA-approved drug and may not pose any fetal risk, this might be an appropriate standard of care until suitable vaccines have been thoroughly developed and tested. In the future, our humanized *in vivo* model incorporating hiNSCs could be utilized to test the effects of other anti-ZIKV therapeutics and could be further adapted to understand the pathophysiology of other infectious agents such as CMV or *Toxoplasma gondii*, which are known to disrupt brain development upon exposure during pregnancy (Cordeiro et al., 2015).

## MATERIALS AND METHODS

### Generation of hiNSCs

hiNSCs were generated as previously described (Cairns et al., 2016). Briefly, human foreskin fibroblasts (HFFs) were plated at a concentration of 10^5^ cells in one gelatin-coated well of a 6-well plate, and cultured in fibroblast media (DMEM, 10% FBS, and 1% antibiotic-antimycotic). Concentrated aliquots of the polycistronic lentivirus expressing OCT4, KLF4, SOX2, and cMYC (Addgene #24603, a gift from Jose Cibelli, Michigan State University) were used to infect the cells in fibroblast medium with polybrene (Millipore) at a multiplicity of infection (MOI)=1-2. Media was changed to hiNSC media: Knockout (KO) DMEM supplemented with 20% KO xeno-free serum replacement, 20 ng/ml recombinant bFGF, 1% Glutamax, 1% antibiotic-antimycotic, and 0.1 mM β-mercaptoethanol which also contained 1% KO growth factor cocktail (GFC) (Invitrogen). Four days later, cells were trypsinized and re-plated onto mouse embryonic fibroblast (MEF) feeder layers previously inactivated by mitomycin C. hiNSC media (without KO-GFC) was subsequently changed every 1-3 days. At day 30 or later, colonies were mechanically picked and passaged onto fresh feeder MEF plates. Each colony represents one hiNSC line. hiNSCs were enzymatically passaged as colonies using trypsin-like enzyme, TrypLE (Invitrogen), expanded, and subsequently frozen to make stocks. All generated lines tested negative for mycoplasma contamination.

### hiNSC differentiation, ZIKV infection and Niclosamide treatment

hiNSC colonies were trypsinized off of MEF feeder layers using TrypLE (Invitrogen), then dissociated by manual pipetting. Cell suspensions were passaged through a 40-70 μM cell strainer to remove larger aggregates. Dissociated hiNSCs were cultured on gelatin-coated plates in Neurobasal media supplemented with 2% B27 (Invitrogen), 1% Glutamax, and 1% antibiotic-antimycotic. For ZIKV infection, we used Zika virus strain MR-766 (GenBank Accession No. KU720415), which was originally isolated from a rhesus monkey in Uganda in 1947, and propagated in the Vero cell line. We purchased culture fluid from ZeptoMetrix (Franklin, MA, USA) and used it to directly infect hiNSCs at a range of MOIs (0.0005–0.05). For mock infections, an equal volume of control 199 culture medium was used. All virus work was approved by Tufts Institutional Biosafety Committee. For Niclosamide (NIC) treatment, NIC (Millipore) powder was reconstituted with 1:1 methanol:acetone at a concentration of 10 mg/ml. This stock concentration was diluted using culture media, and added directly to cells.

### Isolation and culture of embryonic chick cells

To isolate chick cortical neurons, intact brains were dissected from E9 chick embryos, minced with microscissors, then subjected to Trypsin-EDTA 0.25% (Invitrogen) digestion for 10 min at 37°C. Digested cells were pipetted and passed through a 70 μM filter before culture in Neurobasal media supplemented with 2% B27 (Invitrogen), 1% Glutamax, and 1% antibiotic-antimycotic on gelatin-coated plates. Chick dermal fibroblasts were isolated using a similar protocol, with starting material harvested from dorsal skin of E9 embryos. Fibroblasts were cultured in DMEM, 10% FBS, and 1% antibiotic-antimycotic. All studies were conducted in accordance with NIH and Tufts guidelines for embryonated chicken eggs.

### Cytokine array

hiNSCs were mock- or ZIKV-infected at a MOI of 0.005. After 4 days of culture, supernatants were collected and passaged through a low protein binding 0.45 µM filter (Millipore) to remove residual cells and debris. Human Cytokine Array C5 (AAH-CYT-5) was purchased from Raybiotech (Norcross, GA, USA) and was performed according to manusfacturer's instructions. Blots were analyzed using Syngene gel imaging and analysis system (Frederick, MD, USA). Intensity was quantified using ImageJ (NIH) and relative protein expression was determined using Raybiotech software.

### ZIKV ELISA kit

NS1 ELISA kit was purchased from BioFront Technologies (Tallahassee, FL, USA). *In vitro* cell culture supernatants were subjected to ELISA assay according to manufacturer's instructions.

### Immunofluorescence

Cells grown in tissue culture plates were fixed in 4% paraformaldehyde, and washed with 1× phosphate-buffered saline (PBS). Samples were incubated with blocking buffer, which consisted of PBS containing 10% goat serum and 0.1% triton X-100. Primary antibodies were added to blocking buffer, and incubated with samples overnight at 4°C. The next day, samples were washed several times with PBS, and incubated with a corresponding fluorescently conjugated secondary antibody in blocking buffer for 1 h at room temperature. Nuclei were counterstained with DAPI (Invitrogen). For immunostaining samples from *in vivo* studies, 4% paraformaldehyde-fixed cryosectioned tissues were used following a similar immunostaining protocol. Embryos to be cryosectioned were first equilibrated in 15% sucrose-PBS solution, then embedded in OCT. Sections of 10 μM thickness were prepared on slides using a cryostat (Leica). All antibodies used in this study are listed in Table S2.

### qRT-PCR

Total RNA was isolated using the RNeasy Mini kit (Qiagen). cDNA was generated using MLV-reverse transcriptase (Invitrogen, CA, USA) according to the manufacturers' protocols. Quantitative RT-PCR was performed using the CFX96 Real-Time PCR Detection System (BioRad) and normalized against the housekeeping gene GAPDH. All primer sequences are listed in Table S3.

### Injection of hiNSCs into chick embryos

hiNSCs were trypsinized off of MEF feeder layers using TrypLE (Invitrogen), and subsequently dissociated to achieve a single cell suspension. Dissociated hiNSCs were subjected to either mock or ZIKV-infection at a MOI of 0.05. Hamburger Hamilton Stage 16 (∼55 h of incubation) chicken embryos (UConn) were used. A small window was first made in the eggshell to access the embryo. Fast green dye (1 μl) was added to the cell suspension to visualize the location of the injected cells. Cells entered a pulled borosilicate glass needle by capillary action, and were subsequently injected into the lumen of the developing chick encephalon using a micromanipulator (Parker Picospritzer II). PBS with antibiotic-antimycotic was added to prevent infection, and the windowed egg was then sealed using tape. Embryos were allowed to grow in a 37°C chamber for ∼10 days before harvest and fixation with 4% paraformaldeyde for subsequent analysis.

### Systemic *in ovo* ZIKV infection and drug treatment

For systemic virus infection, we initially tested a range of virus particles and found that higher than 20 viral particles/embryo resulted in massive early embryonic death and did not allow for survival of embryos to E12. As such, we infected E5 embryos with 20 viral units/egg in 1 ml PBS with antibiotic-antimycotic applied directly to the CAM. An appropriate dosage for *in vivo* treatment was determined as follows. The weight of an E3 chick embryo is approximately 20 mg. Dosage per egg was 1 µg per egg, which corresponds to a dosage of 50 mg/kg. A stock concentration of 10 mg/ml was generated by reconstituting NIC in methanol:acetone, 1:1. NIC or vehicle was diluted 1 µl/10 ml in PBS with antibiotic-antimycotic and applied directly to the CAM.

### Imaging

Fluorescent images were obtained using a Keyence BZ-X700 microscope and associated software. Larger scale images of embryos were taken with a Samsung Galaxy Camera 2.

### Statistics

All data are expressed as mean±s.d., with at least 3 biological replicates analyzed per experiment. Independent experiments were repeated three times. Data with statistically significant differences were determined by 1-factor ANOVA with post hoc Tukey test using the statistics software SYSTAT12 (Systat). A *P*-value less than 0.05 was considered significant.

## Supplementary Material

Supplementary information
