# Supplementary material for: Niclosamide rescues microcephaly in a humanized in vivo model of Zika infection using human induced neural stem cells
Source: Biol Open. 2018 Jan 15;7(1):bio031807. doi: 10.1242/bio.031807 (PMC5829514; doi:10.1242/bio.031807)
Supplement: Supplementary information [file biolopen-7-031807-s1.pdf]

© 2018. Published by The Company of Biologists Ltd | Biology Open (2018): doi:10.1242/bio.031807: Supplementary information

FIRST PERSON

First person – Dana Cairns

First Person is a series of interviews with the first authors of a selection of papers published in Biology Open, helping early-career researchers promote themselves alongside their papers. Dana Cairns is first author on 'Niclosamide rescues microcephaly in a humanized *in vivo* model of Zika infection using human induced neural stem cells', published in BiO. Dana is a Postdoctoral Scholar in the lab of David L. Kaplan in the Biomedical Engineering Department at Tufts University, USA, guiding projects using human induced neural stem cells to generate complex innervated human tissue-engineered constructs for *in vitro* disease modeling and drug discovery.

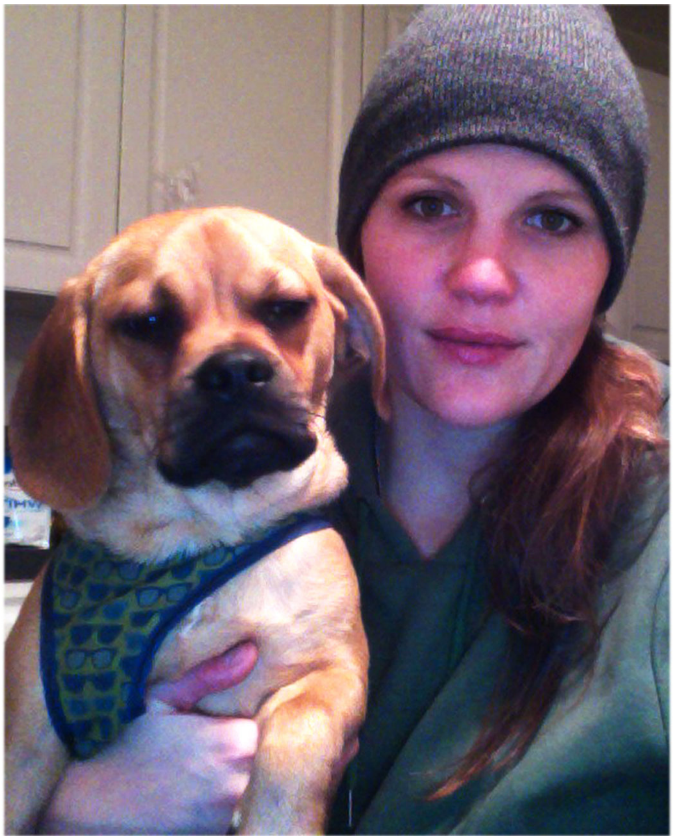

Dana Cairns

What is your scientific background and the general focus of your lab?

I received my PhD in cell, molecular and developmental biology, but my postdoctoral lab focuses more on the generation of novel biomaterials and tissue-engineered models. During graduate school, I initially worked on projects to understand the complex patterning and transcriptional mechanisms that take place during cartilage, bone and muscle development in early stage embryos. As I've transitioned into my postdoc position, I've been able to utilize developmental concepts and mechanisms to guide various tissue engineering projects. I developed a technique for the generation of expandable and rapidly differentiating human induced neural stem cell (hiNSC) lines, which have since been used to establish a variety of innervated human tissue models as well as for the purpose of creating humanized *in vivo* models of disease.

How would you explain the main findings of your paper to non-scientific family and friends?

By now, most people are familiar with the dangers associated with the mosquito-borne Zika virus, in particular with regard to its effects on pregnant women due to its causative link to microcephaly, a congenital birth defect in which babies are born with abnormally small heads and deficits in brain development. Understanding how this virus elicits its detrimental effects using physiologically relevant *in vivo* models is crucial to developing potential strategies to treat and/or prevent this from happening. We injected hiNSCs into developing chick embryo brains, subjected them to Zika infection, and found that these humanized Zika-infected embryos developed severe microcephaly. The chick embryo is a perfect system for this type of work as you can break the shell and add human cells at a very early stage and allow it to continue developing while it is still in the egg, which is something you cannot do in mammalian systems. We show that in the absence of intracranially injected hiNSCs, the resulting embryonic phenotype is unremarkable, suggesting that our humanized *in vivo* model provides a very useful tool for studying neurodevelopmental diseases specific to humans. Lastly, we show that treatment with Niclosamide, an FDA-approved drug used for treating tapeworm infestations, results in a reversal of Zika-induced microcephaly in this humanized model.

Dana Cairns' contact details: Biomedical Engineering Department, Tufts University, Medford, USA.
E-mail: dana.cairns@tufts.edu

"[…] the finding that these embryos developed such severe microcephaly reminiscent of what is seen in human patients was incredibly surprising."

What are the potential implications of these results for your field of research?

From a translational standpoint, this humanized *in vivo* model of Zika-induced microcephaly using hiNSCs could be utilized to validate other Zika treatments and could be further adapted to understand the pathophysiology of other infectious agents known to disrupt brain development upon exposure during pregnancy. Future humanized *in vivo* models could also be developed utilizing hiNSCs generated from specific patient populations to help understand genetic-based models of human neurodevelopmental disease. There are also clinical implications in that we show that Niclosamide, a drug already used clinically to treat unrelated conditions, shows efficacy in promoting survival and rescuing microcephaly and brain abnormalities in humanized Zika-treated embryos.

What has surprised you the most while conducting your research?

Our goal in this study was to develop a humanized *in vivo* model of Zika infection, and the finding that these embryos developed such

Biology Open (2018): doi:10.1242/bio.031807: Supplementary information

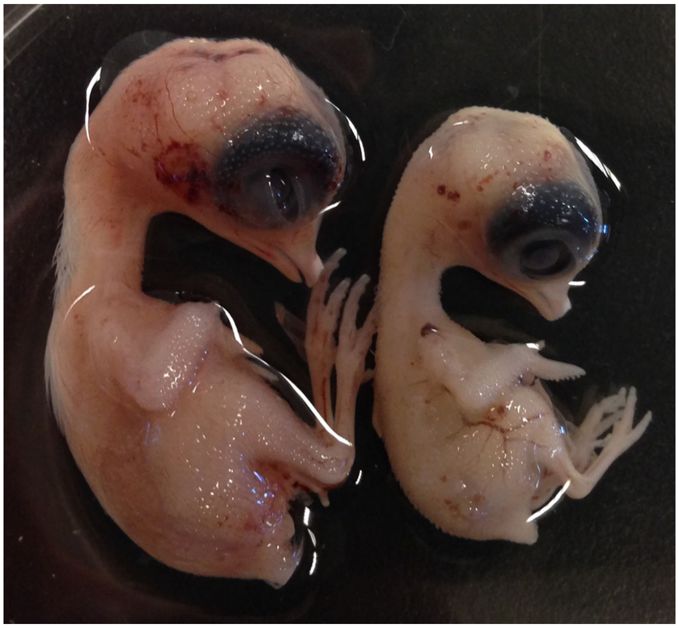

Chick embryos injected with mock- (left) or Zika virus-infected (right) hiNSCs 10 days post-injection.

severe microcephaly reminiscent of what is seen in human patients was incredibly surprising, especially given how comparatively unresponsive other animal models are to systemic Zika infection (including 'non-humanized' or standard chick embryo models, which demonstrate no obvious phenotypic changes in response to Zika infection). It is perhaps in doing these types of human xenograft experiments that we can start to understand why humans are so susceptible to certain diseases while other organisms are not.

What changes do you think could improve the professional lives of early-career scientists?

I think it might be helpful for early-stage scientists to take time during their graduate and post-graduate training to figure out what they like or dislike about their respective fields, and to seek out guidance of specific mentors to help better shape their ultimate career goals. Not everyone who enters graduate school wants to necessarily pursue a traditional academic track; however, many are often unaware of the variety of professional opportunities available to PhD scientists. Over the years, I have seen more and more efforts being made to promote some of these alternative pathways; however, I still think there is some confusion as to how to acquire the different skill sets necessary to obtain these types of positions.

What's the best piece of advice you have received from a mentor?

I had a mentor once tell me that when things in the lab are working really well is when you sort of 'ride the wave of positive results' and work extra hard. When experiments seem to be going badly is when it becomes important to take a step back, and to do something completely different so that you can eventually return to the project with fresh eyes. The notion of taking a break is often lost on those of us at the bench, but maintaining a healthy work-life balance is also essential.

What's next for you?

Hopefully obtaining future employment where I feel as challenged and excited by what I work on as I am now!

Reference

Cairns, D. M., Boorgu, D. S. S. K., Levin, M., Kaplan, D. L. (2018). Niclosamide rescues microcephaly in a humanized *in vivo* model of Zika infection using human induced neural stem cells. *Biol. Open* **7**, doi:10.1242/bio.031807.
